# Employing Genomic Tools to Explore the Molecular Mechanisms behind the Enhancement of Plant Growth and Stress Resilience Facilitated by a *Burkholderia* Rhizobacterial Strain

**DOI:** 10.3390/ijms25116091

**Published:** 2024-05-31

**Authors:** Yueh-Long Chang, Yu-Cheng Chang, Andi Kurniawan, Po-Chun Chang, Ting-Yu Liou, Wen-Der Wang, Huey-wen Chuang

**Affiliations:** 1 Department of Agricultural Biotechnology, National Chiayi University, Chiayi 600355, Taiwan; 2 Department of Agronomy, Brawijaya University, Malang 65145, Indonesia

**Keywords:** microbial IAA, nitrogen fixation, microbial osmolytes, auxin signaling, ethylene synthesis, salicylic signaling

## Abstract

The rhizobacterial strain BJ3 showed 16S rDNA sequence similarity to species within the *Burkholderia* genus. Its complete genome sequence revealed a 97% match with *Burkholderia contaminans* and uncovered gene clusters essential for plant-growth-promoting traits (PGPTs). These clusters include genes responsible for producing indole acetic acid (IAA), osmolytes, non-ribosomal peptides (NRPS), volatile organic compounds (VOCs), siderophores, lipopolysaccharides, hydrolytic enzymes, and spermidine. Additionally, the genome contains genes for nitrogen fixation and phosphate solubilization, as well as a gene encoding 1-aminocyclopropane-1-carboxylate (ACC) deaminase. The treatment with BJ3 enhanced root architecture, boosted vegetative growth, and accelerated early flowering in *Arabidopsis*. Treated seedlings also showed increased lignin production and antioxidant capabilities, as well as notably increased tolerance to water deficit and high salinity. An RNA-seq transcriptome analysis indicated that BJ3 treatment significantly activated genes related to immunity induction, hormone signaling, and vegetative growth. It specifically activated genes involved in the production of auxin, ethylene, and salicylic acid (SA), as well as genes involved in the synthesis of defense compounds like glucosinolates, camalexin, and terpenoids. The expression of AP2/ERF transcription factors was markedly increased. These findings highlight BJ3’s potential to produce various bioactive metabolites and its ability to activate auxin, ethylene, and SA signaling in *Arabidopsis*, positioning it as a new *Burkholderia* strain that could significantly improve plant growth, stress resilience, and immune function.

## 1. Introduction

Employing beneficial microorganisms to promote plant growth and reduce stress damage has become a valuable strategy in agricultural practice. Known as plant-growth-promoting rhizobacteria (PGPR), these beneficial microbes in the rhizosphere significantly impact plant health and productivity. PGPR can enhance plant growth by producing phytohormones such as indole acetic acid (IAA), a natural form of auxin, which is an essential regulator of lateral root formation [1]. Rhizobacteria may increase nutrient availability for plant growth through their activities of mineral solubilization, siderophore production, and nitrogen fixation [2]. Alternatively, PGPR enhance plant growth vigor through the production of various antimicrobial metabolites that suppress the viability of pathogens [3]. As a biocontrol agent, PGPR not only directly target pathogens but also produce elicitors that enhance a plant’s innate defenses, a response known as induced systemic resistance (ISR). In this defense mechanism, jasmonic acid (JA) and ethylene signals are crucial for reshaping plant metabolism to build resistance against pathogens [4]. ISR, elicited by PGPR, exhibits phenotypic similarities to pathogen-induced systemic acquired resistance (SAR), in which salicylic acid (SA) acts as a key regulator for stimulating cellular pathways to establish disease resistance [5]. In the natural environment, plants interact with rhizobacteria by using their pattern recognition receptors (PRRs) to recognize pathogen-associated molecular patterns (PAMPs), also referred to as microbial-associated molecular patterns (MAMPs) [6]. PRRs recognize the PAMPs/MAMPs and subsequently activate PAMP/MAMP-triggered immunity (PTI/MTI), conferring an effective defense response [7].

PGPR produce various types of bioactive metabolites, which can exert multifaceted effects to improve plant tolerance to unfavorable environments. Throughout their life cycle, plants face a range of environmental stresses at various developmental stages. Root systems are crucial for the uptake of nutrients and water, making them vital for a plant’s survival. Consequently, the root system is key to ensuring that the aboveground parts of the plant can adapt to environmental challenges. Lateral roots are important to expand the root system for nutrient acquisition. Auxin is a key regulator of root growth and development [1]. PGPR are known to stimulate root architecture in the host plant by producing significant amounts of IAA [8]. In addition to its main function in controlling plant growth, studies have shown the regulatory role of auxin in plant responses to abiotic stress. For example, auxin biosynthesis and transport are altered in plants under salt stress conditions [9,10]. Additionally, PGPR can enhance the synthesis of IAA in wheat under salt and drought stress conditions [11]. Moreover, treating banana plants with a *Pseudomonas* PGPR strain not only increased their resistance to drought and submergence stress but also activated genes linked to the auxin signaling pathway [12]. As plants encounter an unfavorable environment, ethylene is produced to reduce their growth and proliferation [13]. Studies indicate that PGPR mitigate the adverse effects of stressful conditions by producing ACC deaminase, which in turn reduces ethylene production in the host plants [14]. Ethylene suppresses the development of lateral roots by impacting the polar transport of auxin [15]. However, in collaboration with auxin, ethylene also actively promotes the formation of root hairs [16]. Root hairs, components of the root architecture system, play a role in the adaptive response to abiotic stress [17]. For example, under conditions of phosphate starvation, ethylene signaling increases, resulting in enhanced root hair formation [18]. Moreover, ethylene collaborates with JA to regulate the *AP2/ERF* transcription factor gene family, thereby exerting a positive effect on plant development by enhancing tolerance to abiotic stress and disease resistance [19]. SA and JA signals play crucial roles in triggering plant immunity against pathogens. Additionally, both SA and JA can mitigate the adverse effects of abiotic stress on plant growth by activating antioxidant defenses [20]. For example, SA is a key regulator of the 2,3-butanediol VOC-induced drought stress tolerance [21]. Volatile compounds from a *Bacillus* strain induced salt stress tolerance in *Arabidopsis* due to the activation of the JA signaling pathway [22].

The bacterial strains of the *Burkholderia* genus are widely distributed in the soil. These bacterial strains promote plant growth through their phosphate-solubilizing activities [23], nitrogen-fixation ability [24], and altering phytohormone signaling in the host plants [25]. Genes involved in the synthesis of various antimicrobial metabolites have been identified in the *Burkholderia* genome [26]. Several strains of the *Burkholderia contaminans* species have demonstrated their effectiveness in biocontrol of plant diseases [27,28]. *Burkholderia phytofirmans* PsJN has been reported to activate induced resistance against gray mold and to enhance cold stress tolerance by increasing the levels of stress-related metabolites in a low-temperature environment [29].

In this study, the bacterial strain BJ3 was identified as a newly isolated *Burkholderia contaminans* strain based on its whole-genome sequencing results. Gene clusters associated with plant-growth-promoting traits (PGPTs) were identified; among these, genes involved in the synthesis of IAA, osmolytes, NRPS, and VOCs were particularly significant. This genome also contains genes related to nitrogen fixation and those encoding ACC deaminase. BJ3 not only promoted vegetative growth but also stimulated an early flowering phenotype in the treated plants. Moreover, this treatment improved plant tolerance to drought and salt stress. A transcriptome analysis revealed that BJ3 treatment activated the expression of genes associated with stress tolerance and vegetative growth, as well as genes linked to hormone signaling pathways, including auxin, ethylene, and SA.

## 2. Results

### 2.1. Molecular Characterization of BJ3

**Figure 1:**
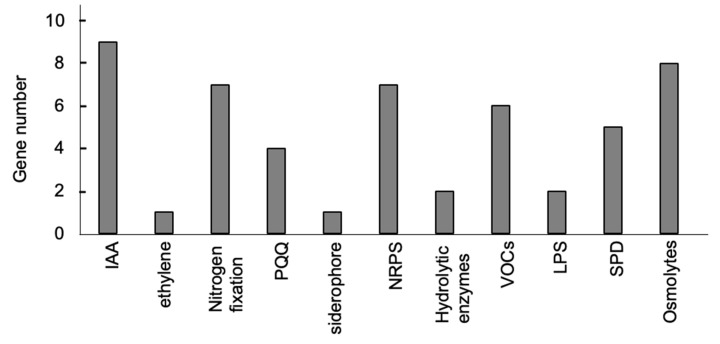
Gene clusters associated with plant-growth-promotion traits. The BJ3 genome contains candidate genes that function in promoting plant growth.

The sequencing results for the 16S rDNA PCR fragment from BJ3 bacterial genomic DNA showed 100% identity with several strains of the *Burkholderia* genus, including *Burkholderia contaminans*, *Burkholderia* sp., and *Burkholderia cenocepacia* (Table 1). To identify genes associated with plant growth promotion in the BJ3 genome, the whole genome sequence was analyzed using both Illumina and Nanopore sequencing systems. The results revealed 7361 open reading frames (ORFs) in the BJ3 genome, which were assigned to two chromosomes. Of these, 7112 (96.62%) ORFs showed sequence identity to *Burkholderia contaminans*, while 165 (2.24%) ORFs matched sequences from various *Burkholderia* species. Gene clusters responsible for synthesizing metabolites that function in plant growth promotion were identified and are listed in Table 2. These genes are involved in producing IAA, pyrroloquinoline quinone (PQQ), NRPS, siderophores, volatile organic compounds (VOCs), spermidine (SPD), osmolytes, lipopolysaccharides, and hydrolytic enzymes, such as chitinase and cellulase. The identified PGPT-associated genes also play a role in nitrogen fixation and code for ACC deaminase, which has activity for reducing ethylene synthesis (Figure 1). Microbes producing IAA and ACC deaminase may promote plant growth by altering hormone signaling in the host plants [8,14]. The BJ3 genome contains a large number of genes coding for proteins involved in IAA synthesis via three pathways: indolepyruvate ferredoxin oxidoreductase (IOR) in the indole-3-pyruvate (IPyA) pathway [30], iaaH in the indole-3-acetamide (IAM) pathway [31], and nitrile hydratase (nth) in the indole-3-acetaldoxime/indole-3-acetonitrile (IAOx/IAN) pathway [32]. Gene clusters code for proteins functioning in the regulation of vegetative growth in plants, including nitrogen fixation, the synthesis of pyrroloquinoline quinone (PQQ), and siderophore production. Genes identified in the BJ3 genome coding for proteins with functions related to the nitrogen-fixation process included IscU and Nif3, which exhibit nitrogenase activity [33], along with sigma factor 54-interacting protein and glnA, both playing regulatory roles in nitrogen fixation [34,35]. Pyrroloquinoline quinone (PQQ) is known to play a crucial role in stimulating plant growth and facilitating phosphorous solubilization [36] and is a scavenger of ROS [37]. Furthermore, *PvdF* is involved in the synthesis of siderophore and pyoverdine [38]. A large gene cluster for the synthesis of NRPs/polyketides and VOCs was identified; these metabolites possess antimicrobial activities [39,40,41,42]. Additionally, two genes, chitinase and cellulase, were identified; the antimicrobial activity of these two hydrolytic enzymes has been characterized [43,44]. Genes involved in the synthesis of lipopolysaccharides were discovered in the BJ3 genome. Bacterial lipopolysaccharides can act as elicitors to trigger plant defense responses [45]. Furthermore, five genes involved in the synthesis of spermidine were identified. Extracellular spermidine produced by *Bacillus amyloliquefaciens* SQR9 has been demonstrated to enhance salt stress tolerance in *Arabidopsis* and maize [46]. The BJ3 genome was found to contain genes involved in the synthesis of metabolites capable of regulating osmotic stress; such metabolites included proline, glycine betaine, trehalose, and ectoine. Bacteria that produce these metabolites have been shown to alleviate osmotic stress in plants [47,48,49,50].

### 2.2. Biochemical Analysis of Strain BJ3

**Figure 2:**
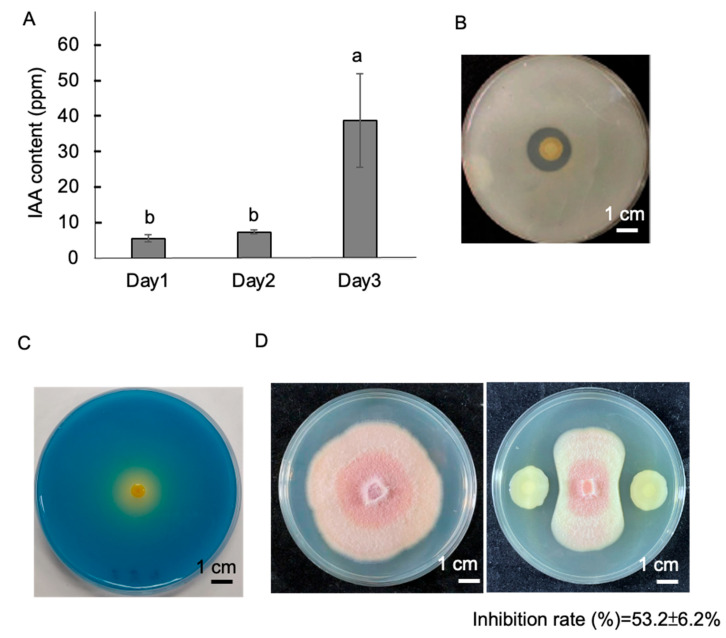
Physiological properties of strain BJ3. The bacterial strain produced IAA; the highest concentration of IAA was detected after a three-day culture period (**A**). Error bars present the SD from the mean of three replicates. Different letters within the histograms represent statistical significance at a *p*-value of 0.05. The phosphate-solubilizing activity of the tested bacterial strain is indicated by the production of an obvious clear zone in the NBRIP medium (**B**). The nitrogen-fixation activity of the tested bacterial strain is indicated by a change in the color of bromothymol blue to yellow (**C**). The tested bacterial strain inhibited the mycelial growth of *Foc* TR4, and the rate of mycelial inhibition is indicated at the bottom of the photo (**D**).

Biochemical analysis indicated that BJ3 produced a higher amount of IAA on the third day of culture, with approximate average concentrations of 38 ppm (Figure 2A). This bacterial strain exhibited significant phosphate-solubilizing activity, generating a clear zone surrounding the bacterial colony in the NBRIP medium (Figure 2B). Nitrogen-fixation activity was analyzed by inoculating BJ3 in the Jensen’s medium, which resulted in the bromothymol blue indicator changing to yellow (Figure 2C). Furthermore, BJ3 also exhibited antifungal activity, suppressing approximately 53% of the mycelial growth of the fungal pathogen *Fusarium oxysporum* f. sp. *cubense* tropical race 4 (*Foc* TR4) (Figure 2D).

### 2.3. Arabidopsis Growth Promotion Stimulated by Strain BJ3

**Figure 3:**
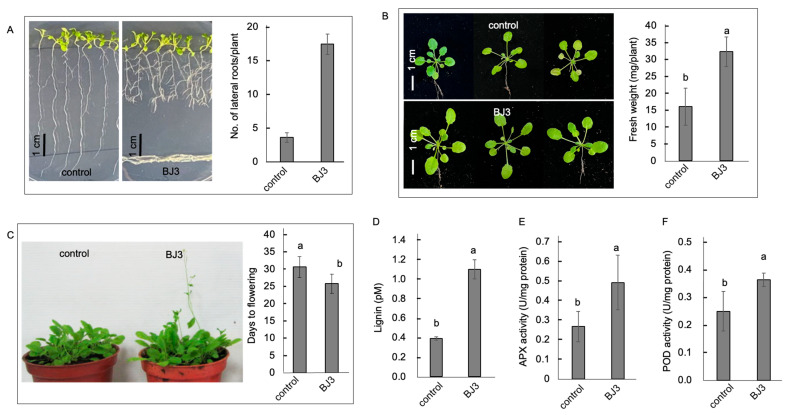
Physiological alterations in *Arabidopsis* seedlings in response to BJ3 treatment. BJ3 inoculation increased the root architecture and lateral root number in seedlings (**A**). The treated seedlings grown in the soil exhibited increased plant size and fresh weight (**B**). An early flowering phenotype was observed, and days to flower development were reduced (**C**). Error bars present the SD from the mean and n = 10. The BJ3 treatment increased lignin content (**D**), as well as the activity of antioxidant enzymes APX (**E**) and POD (**F**). Values in each histogram represent the mean of three replicates ± SD. Different letters within the histograms represent statistical significance at a *p*-value of 0.05.

Metabolites produced by beneficial rhizobacteria can enhance plant growth by affecting root growth [51]. In this study, *Arabidopsis* seedlings cocultured with BJ3 for four days underwent significant changes, as evidenced by an increased number of lateral roots and a reduction in the length of primary roots (Figure 3A). The number of lateral roots in seedlings treated with BJ3 was significantly higher than that in control seedlings (Figure 3A). Furthermore, the soil-grown *Arabidopsis* seedlings treated with BJ3 showed an increase in plant size and fresh weight compared to the control plants (Figure 3B). Three weeks after treatment, the BJ3-treated seedlings displayed visible floral inflorescences earlier than the control seedlings. The days required for the appearance of floral inflorescence in the BJ3-treated plants were significantly fewer than those in the control plants (Figure 3C). Furthermore, seedlings treated with BJ3 exhibited elevated levels of lignin in their leaf tissues (Figure 3D) and increased activity of antioxidant enzymes, such as ascorbate peroxidase (APX) and guaiacol peroxidase (POD) (Figure 3E,F).

### 2.4. BJ3 Treatment Enhanced Seedling Stress Tolerance

**Figure 4:**
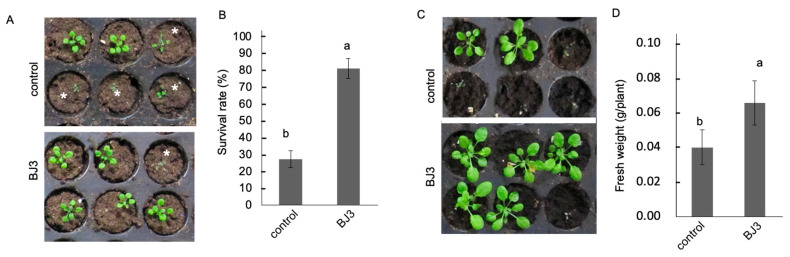
Drought stress tolerance induced by BJ3. After water deprivation, the seedlings treated with BJ3 exhibited a greater number of unwilted individuals (**A**); an asterisk indicates the wilted seedlings. The BJ3-treated seedlings showed a higher survival rate (**B**). Seven days after rewatering following the drought stress treatment, the BJ3-treated seedlings exhibited increases in both plant size (**C**) and fresh weight (**D**). Values in each histogram represent the mean of three replicates ± SD. Different letters within the histograms represent statistical significance at a *p*-value of 0.05.

**Figure 5:**
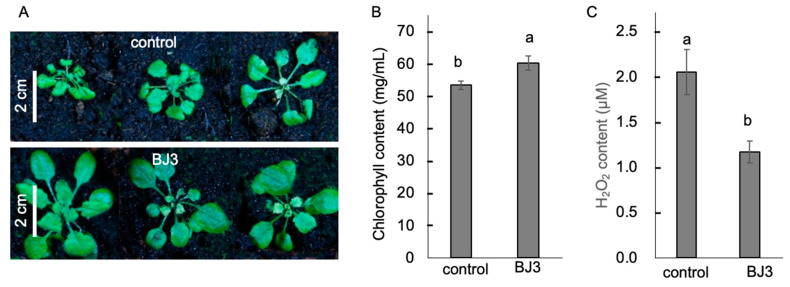
Salt stress tolerance induced by BJ3. Two weeks after the salt stress treatment, the seedlings treated with BJ3 exhibited a larger plant size (**A**), higher chlorophyll content levels (**B**), and reduced H_2_O_2_ accumulation (**C**) compared to the control. Different letters within the histograms represent statistical significance at a *p*-value of 0.05.

Two-week-old *Arabidopsis* seedlings were pre-treated with BJ3 prior to undergoing a five-day period of water deprivation. After the drought stress, the BJ3-treated seedlings exhibited a greater number of unwilted individuals and demonstrated a higher survival rate (Figure 4A,B). Seven days after rehydration in the post-drought period, the BJ3-treated seedlings showed increases in both plant size and fresh weight (Figure 4C,D). Moreover, an analysis of salt stress tolerance was performed in the BJ3-treated seedlings. Two weeks after the salt stress treatment, the seedlings that received the BJ3 treatment demonstrated greater growth in plant size (Figure 5A), elevated levels of chlorophyll content (Figure 5B), and a diminished buildup of H_2_O_2_ (Figure 5C) in contrast to the untreated control group.

### 2.5. BJ3 Altered Arabidopsis Transcription Associated with Plant Development and Stress Response

**Figure 6:**
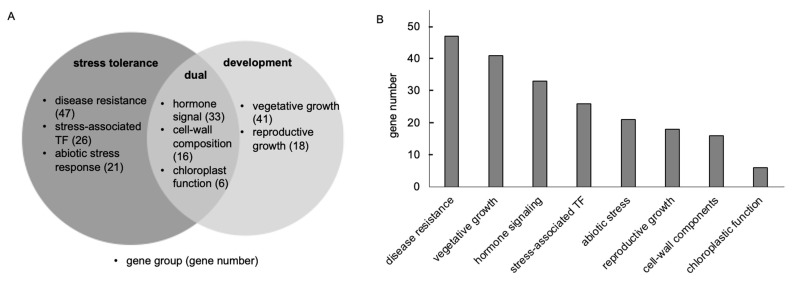
Transcriptomic alterations of Arabidopsis seedlings in response to BJ3 treatment. The BJ3 upregulated genes were classified into eight groups based on their annotated functions; moreover, these groups were assigned to biological functions related to stress tolerance, development, or both (**A**). The number of genes in each group is presented (**B**).

**Figure 7:**
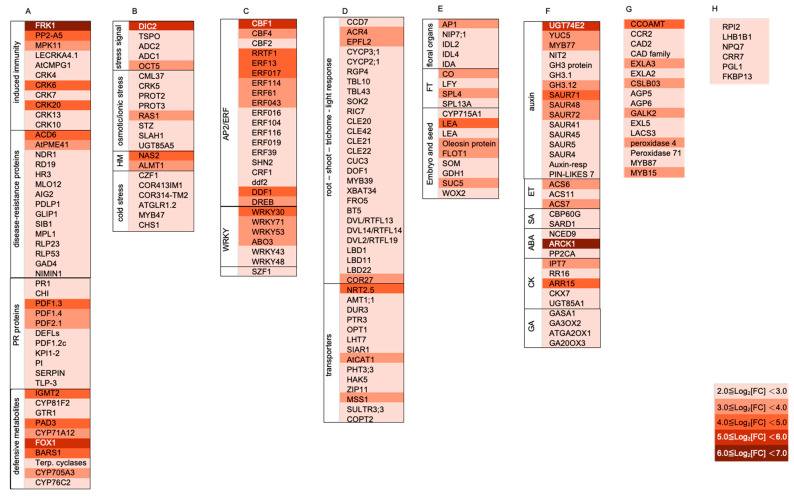
The BJ3 upregulated genes from the Arabidopsis transcriptome analysis. Upregulated genes exhibit annotated functions linked to disease resistance (**A**), abiotic stress (**B**), stress-associated transcription factors (TF) (**C**), vegetative growth (**D**), reproductive growth (**E**), hormone signaling (**F**), cell-wall components (**G**), and chloroplast function (**H**). Different shades of red indicate the fold changes in gene expression.

PGPR exert a strong effect on modulating plant gene expression. To gain insight into the mechanisms involved in plant growth promotion mediated by BJ3, transcriptome alterations of *Arabidopsis* treated with BJ3 were analyzed. In total, 208 *Arabidopsis* genes, which exhibited induction levels greater than Log_2_[FC] = 2.0, were subjected to further analysis. The detailed gene information is listed in Table S1. As shown in Figure 6A, the upregulated genes are associated with cellular pathways that govern stress tolerance. This includes groups of genes related to disease resistance, stress-associated transcription factors (TFs), and the response to abiotic stress. Upregulated genes also play roles in regulating plant development, such as vegetative and reproductive growth. Furthermore, some of these upregulated genes serve multiple purposes, influencing both stress response and cellular growth by altering hormone signaling, the composition of the cell wall, and chloroplast function. Among these genes, those related to the regulation of disease resistance constituted the largest group (Figure 6B). This group included genes encoding signaling molecules, disease-resistance proteins, pathogenesis-related (PR) proteins, and enzymes involved in the synthesis of defense metabolites, such as glucosinolate, camalexin, and terpenoids (Figure 7A). BJ3 strongly induced the expression of *FLG22-INDUCED RECEPTOR-LIKE KINASE 1* (*FRK1*), a marker for the activation of PTI/MTI [52]. Additionally, it upregulated genes such as *MPK11*, *AtCMPG1*, and *LecRKA4.1*, which play various roles in PTI/MTI [53,54,55]. Furthermore, *PP2-A5*, encoding a protein belonging to the Phloem Protein2 family, is involved in the induced immunity against insect damage [56]. BJ3 treatment also increased the expression levels of cysteine-rich receptor-like kinase (CRK) gene members. For example, *Arabidopsis CRK13*’s function is involved in the disease resistance against *Pst* DC3000 and SA accumulation [57]. Another upregulated gene, *ACCELERATED CELL DEATH 6* (*ACD6*), is a regulator of the defense response mediated by SA [58]. BJ3 also induced the expression of *AtPME41*, which encodes a pectin methylesterase functioning in the disease resistance against *P. syringae* [59]. Moreover, Arabidopsis *GDSL lipase 1* (*GLIP1*) plays a role in controlling systemic immunity by regulating components of the ethylene signaling pathway [60]. The major *PR* genes induced by BJ3 included members of the defensin-like family (Figure 7A). BJ3 treatment also activated genes involved in the synthesis of secondary metabolites associated with the defense response, including *IGMT2* and *CYP81F2* for glucosinolate synthesis [61,62]; *CYP71A12* and *PHYTOALEXIN DEFICIENT 3* (*PAD3*) for camalexin synthesis [63,64]; *FAD-linked oxidoreductase 1* (*FOX1*) for cyanogenic phytoalexin synthesis [65]; and four genes encoding enzymes for terpenoid synthesis, which include *BARUOL SYNTHASE 1* (*BARS1*), *terpenoid cyclase* (AT1G48820), *CYP705A3*, and *CYP76C2* [66,67].

In Figure 7B, the upregulated genes displaying annotated functions related to abiotic stress responses are highlighted. These include genes regulating the perception and response to stress signals, as well as tolerance to osmotic/ionic stress, heavy metals, and low temperatures. For example, dicarboxylate carrier 2 (DIC2) is important for shuttling malate-oxaloacetate and maintaining redox balance between the mitochondria and the cytosol; genes of DICs are important for plant adaptation to environmental stresses [68,69]. Genes involved in the synthesis of polyamines (PAs) and PA transport include *ADC1*, *ADC2*, and *OCT5*. PAs are polycations and low-molecular-weight metabolites that can exert protective roles on cellular molecules under increased oxidative stress conditions [70]. Furthermore, the expression of *RESPONSE TO ABA AND SALT 1* (*RAS1*) was increased. *Nicotianamine synthase 2* (*NAS2*) is for the synthesis of nicotianamine (NA), acting as a ligand for the detoxification of heavy metals [71]. *Aluminum-activated malate transporter 1* (*ALMT1*) regulates aluminum tolerance in *Arabidopsis* by secreting malate to chelate the Al in acidic soils [72].

BJ3 upregulated a group of genes encoding transcription factors, playing a dual function in the regulation of abiotic and biotic stress-related physiology (Figure 7C). Among these transcription factors, AP2/ERF transcription factors occupied the largest portion of this gene group (Figure 7C). The expression of *CRT/DRE BINDING FACTOR 1* (*CBF1*), *CBF2*, and *CBF3*, members of the AP2/ERF family, is rapidly induced in response to low temperature [73]. *Redox Responsive Transcription Factor 1* (*RRTF1*), also known as *ERF109*, is an important regulator to control redox homeostasis plant cells after exposure to abiotic stress [74]. *ERF114* mediates elicitor-induced disease resistance in *Arabidopsis* [75]. *DWARF AND DELAYED FLOWERING 1* (*DDF1*) functions in the adaptation response to multiple abiotic stress [76]. Additionally, BJ3 upregulated the expression of several *WRKY* transcription factors. Among them, *WRKY30* plays a dual function in the regulation of disease resistance [77] and abiotic stress tolerance [78]. The regulatory role of *WRKY53* is implicated in leaf senescence and disease resistance against *P. syringae* in *Arabidopsis* [79,80]. Furthermore, *SALT-INDUCIBLE ZINC FINGER 1* (*SZF1*) encodes a CCCH-type zinc finger protein, displaying functions involved in salt stress and immune responses [81].

### 2.6. BJ3 Enhanced Arabidopsis Gene Expression Related to Plant Development

The second-largest group of upregulated genes was associated with various aspects of plant growth (Figure 6B). As shown in Figure 7D, genes regulated by BJ3 were involved in regulating root and shoot growth, trichome development, light response, and nutrient transport. Upregulated genes included those encoding the *CLAVATA3/ESR* (*CLE*) peptides, *Devil/Rotundifolia Like* (*RTFL*) peptides, and *LOB domain-containing proteins* (*LBD*). The gene function of the *CLE* family acts as a signaling molecule mediating the *WUSCHEL* (*WUS*) signaling pathway, which is implicated in maintaining the shoot apical meristem (SAM) [82]. The *RTFL* gene family consists of small peptides that regulate development in *Arabidopsis* [83]. *LBD* gene members encode plant-specific transcriptional regulators that play roles in determining organ boundaries during plant development [84]. Another upregulated gene, *EPIDERMAL PATTERNING FACTOR-like protein 2* (*EPFL2*), also plays a role in controlling shoot organ boundaries [85]. The function of *Arabidopsis Crinkly4 Related 4* (*ACR4*) is implicated in regulating root-cell division [86]. *MYB39* is involved in regulating suberin biosynthesis in the root endodermis of *Arabidopsis* [87]. Moreover, *COLD REGULATED GENE 27* (*COR27*) plays a role in controlling the circadian rhythm of plant growth [88]. BJ3 also stimulated the expression of several transporters involved in nitrogen metabolism, including *Nitrate transporter 2.5* (*NRT2.5*), *Ammonium transporter 1 member 1* (*AMT1;1*), *Urea-proton symporter* (*DUR3*), *PEPTIDE TRANSPORTER 3* (*PTR3*), *Oligopeptide transporter 1* (*OPT1*), *Lysine histidine transporter-like 7* (*LHT7*), and *Siliques Are Red 1* (*SIAR1*). Among them, the induction fold of *NRT2.5* in response to BJ3 treatment was the highest in this gene group, with a Log_2_[FC] of 4.9.

Additionally, upregulated genes constituted several genes showing annotated functions linked to the regulation of floral organ development, flowering time, and embryo and seed development (Figure 7E). For example, *APETALA 1* (*AP1*) affects floral organ development [89]. The gene function of *INFLORESCENCE DEFICIENT IN ABSCISSION* (*IDA*) and *INFLORESCENCE DEFICIENT IN ABSCISSION* (*IDA*)-*LIKE* (*IDL*) is to regulate floral organ abscission [90]. The transcription factors *CONSTANS* (*CO*), *LEAFY* (*FLY*), and *squamosa-promoter binding protein-like* (*SPL*) are involved in the regulation of flowering time [91,92]. Lea and Oleosin proteins are important during seed maturation [93].

### 2.7. BJ3 Activated Genes with Multiple Functions in Plant Growth and Stress Tolerance

Rhizobacteria have an impact on plant growth and stress tolerance by influencing the signaling of plant hormones [94]. As shown in Figure 7F, the BJ3 treatment activated the expression of genes involved in hormone signaling transduction pathways, including those for auxin, ethylene, abscisic acid (ABA), SA, gibberellin (GA), and cytokinin (CK). Of these, the genes related to auxin synthesis and response formed the most significant group. The expression of *UGT74E2*, responsible for the production of indole-3-butyric acid (IBA) glucose conjugates [95], was highly induced, with an induction fold of Log_2_[FC] greater than 5. Additionally, upregulated genes included *YUC5*, encoding a flavin monooxygenase that catalyzes the conversion of indole-3-pyruvate (IPA) to IAA, and *NIT2*, encoding a nitrilase involved in the last step of the synthesis of *IAA* [96,97]. Furthermore, upregulated genes also included early auxin response genes and *SAUR* and *GH3* gene members [98]. The transcription factor *MYB77* is involved in the regulation of auxin signaling [99]. In the seedlings treated with BJ3, three upregulated genes encoding 1-aminocyclopropane-1-carboxylic acid (ACC) synthase (ACS) proteins were identified, ACS6, ACS7, and ACS11, all of which are involved in the synthesis of ethylene. Additionally, genes associated with SA biosynthesis and response, such as *SARD1* and *CBP60G* [100], were identified. BJ3 treatment activated the expression of genes involved in ABA synthesis, such as *NCED9* [101], and negative regulators of ABA signal transduction, such as *ARCK1* and *PP2CA* [102,103]. The treatment also upregulated genes involved in the cytokinin and GA signaling pathways. However, genes involved in the biosynthesis and degradation of GA and cytokinin had increased expression levels. These results suggested that BJ3 treatment exerted positive effect on auxin, ethylene, and SA signaling in *Arabidopsis*.

During various developmental stages, the structural integrity of the cell wall requires constant adjustments to facilitate plant growth [104]. As shown in Figure 7G, the analysis of transcriptome alterations revealed a group of upregulated genes that show functions associated with the synthesis of metabolites to fortify the cell wall. For example, *Caffeoyl-CoA O-methyltransferase* (*CCoAMT*), *Cinnamoyl-CoA reductase 2* (*CCR*), and *Cinnamyl alcohol dehydrogenase 2* (*CAD2*) encode proteins that are involved in lignin synthesis [105]. The transcription factors MYB87 and MYB15 are regulators of cell-wall strengthening [106,107]. Chloroplasts play a crucial role in energy production via photosynthesis and oxygen release, both of which promote plant growth and enhance crop yields. Additionally, chloroplasts produce biologically active metabolites that enable plants to adapt and respond to stress from harsh environments [108]. As shown in Figure 7H, a gene encoding ribose-5-phosphate isomerase (RPI2) is required for maintaining the chloroplast structure and its photosynthetic capacity [109], and 6-Phosphogluconolactonase 1 (PGL1) is a key enzyme in the oxidative pentose phosphate pathway (OPPP). Moreover, the PGL genes are responsive to abiotic stresses in rice [110].

## 3. Discussion

The application of *Burkholderia contaminans* strains for biocontrol purposes has been emphasized, which involves the production of diffusible or volatile antimicrobial metabolites [27,28,111]. In this study, the BJ3 genome contains gene clusters involved in the synthesis of diverse metabolites with antimicrobial activity; among these, NRPS/polyketides and VOCs constitute the two largest groups. BJ3’s metabolites demonstrated significant antifungal activity in suppressing the banana Fusarium wilt pathogen. According to a previous study, the expression of non-ribosomal peptide synthetases in the *Burkholderia contaminans* strain MS14 is essential for its antifungal activity against *Geotrichum candidum* [112]. Another study has shown that terpenes, produced by the *Burkholderia gladioli* strain, are identified as effective antifungal metabolites against *Fusarium oxysporum* [113]. In addition to exhibiting antifungal activity, BJ3 treatment increased the expression of a large number of *Arabidopsis* genes linked to cellular pathways associated with induced immunity. BJ3 strongly activated the expression of *FRK1*, a marker gene for PTI/MTI [52], and *CRK6* and *CRK20*, regulators of the PTI/MTI response [114,115]. Moreover, the BJ3-upregulated genes encode disease-resistance proteins, PR proteins, and those for the synthesis of antimicrobial metabolites, such as glucosinolates, camalexin, and terpenoids [116]. These results illustrate the ability of BJ3 to serve a biocontrol purpose.

Genes involved in the synthesis of IAA constitute the largest gene group associated with plant-growth-promoting traits in the BJ3 genome. IAA is the most abundant form of auxin. This phytohormone plays a crucial role in the regulation of lateral root development [1]. BJ3 produced a significant amount of IAA (close to 40 ppm) and induced the *Arabidopsis* gene expression involved in auxin synthesis and response. A consistent phenotype has been observed, showing that treatment with BJ3 significantly enhances lateral root development in *Arabidopsis* seedlings. The BJ3 genome contains gene clusters involved in nitrogen fixation and phosphate solubilization; consistently, BJ3 exhibits significant activity in both processes. These microbial activities may contribute to the growth-promoting effects derived from BJ3. Additionally, *Arabidopsis* treated with BJ3 exhibited an early flowering phenotype. This early flowering is consistent with the activated expression of the *Arabidopsis* genes *CO*, *LFY*, and *SPL13A*, which are involved in regulating the transition from vegetative growth to flower initiation [117,118,119]. Ethylene is a regulator for stimulating the transition of vegetative growth to floral development in *Arabidopsis* [120]. In the BJ3-treated *Arabidopsis* transcriptome, upregulated genes include three members of the ACS family, which is the rate-limiting enzyme in ethylene synthesis [121]. Further evidence supporting an increased ethylene signal in BJ3-treated *Arabidopsis* is that a large number of *AP2/ERF* gene members are stimulated in their expression by BJ3. The expression of these transcription factors is manipulated by the ethylene signal and is crucial for the plant’s adaptive response to various abiotic stresses [122]. Our results indicate that BJ3 activates both auxin and ethylene signals to alter plant growth and development. These findings are consistent with a study demonstrating that these two hormonal signals play roles in plant growth induced by *Burkholderia phytofirmans* [123].

The BJ3 genome contains a notable number of genes involved in the synthesis of various osmolytes, which may regulate the osmotic balance in plants under osmotic stress conditions [47,48,49,50]. Consistently, the BJ3-treated *Arabidopsis* exhibited increased tolerance to both drought and salt stress. In addition to producing metabolites that act as osmolytes to protect plants from oxidative damage under osmotic/salt stress, microbes can also activate the plant antioxidant defense system to reduce the production of ROS under stressful conditions [124]. The BJ3 genome includes a gene cluster dedicated to producing spermidine. Strains of PGPR that produce spermidine have been shown to enhance salt stress tolerance in host plants [46]. Additionally, applying spermidine externally can stimulate antioxidant activity in plants [125]. Moreover, BJ3 contains genes for the synthesis of terpenoids and activates *Arabidopsis* genes related to terpenoid synthesis. This natural compound, in addition to serving as an antimicrobial agent, can also act as an antioxidant in plants [126]. BJ3 treatment triggered the expression of genes involved in SA biosynthesis and signaling pathways. SA plays a beneficial role in enhancing plant tolerance to various abiotic stresses through the activation of antioxidant activity [127]. In addition to playing a crucial role in the regulation of root development, a regulatory role of auxin in stress adaptation is revealed. For example, transgenic potato overexpressing the auxin biosynthesis gene *YUCCA6* resulted in an increased auxin level and enhanced drought stress tolerance [128]. Moreover, auxin improves plant abiotic stress tolerance by manipulating antioxidant activity in plant cells [129]. In this study, BJ3 treatment activated antioxidant enzymes and enhanced plant tolerance to both drought and salt stress. Therefore, BJ3 may serve a protective function in plants, either by producing various types of osmolytes or by activating signaling pathways that help develop an adaptive response to stress.

## 4. Materials and Methods

### 4.1. Bacterial Strain Characterization

The BJ3 strain was isolated from a growing substrate. PCR amplification of the genomic DNA from the bacterial strain was performed using the primers fD1 (5′AGAGTTTGATCCTGGCTCAG3′) and rP1 (5′ACGGTTACCTTGTTACGACTT3′), targeting the 16S rDNA sequence [130]. The PCR fragment was analyzed using a 3730 DNA Analyzer (Applied Biosystems^®^; Foster City, CA, USA). The obtained 16S rDNA sequences were analyzed using the Basic Local Alignment Search Tool (BLAST) program [131] in the NCBI database (https://blast.ncbi.nlm.nih.gov/Blast.cgi (accessed 1 September 2023)).

### 4.2. De Novo Whole Genome Assembly

The fragmented genome DNA of BcD1 was prepared using the Celero PCR workflow with an Enzymatic Fragmentation DNA-Seq Kit (Tecan Trading AG, Männedorf, Switzerland). DNA sequencing was performed by the paired-end method on the Illumina MiSeq system (Illumina, Inc., San Diego, CA, USA). The raw sequence data’s quality was assessed with NanoPlot v1.28.1 [132]. Following this, adapters, low-quality sequences (Q20), and ambiguous bases were trimmed from the reads. The de novo assembly of the genome sequencing data was carried out using SPAdes v.3.14.1 [133]. Open reading frames (ORFs) were predicted by GlimmerHMM [134]. RNAmmer and tRNAscan SE were used to predict rRNA and tRNA, respectively [135,136]. Gene sequences were annotated through the NCBI database. The functional analysis of the genes was performed using FastAnnotator [137] and the Gene Ontology Consortium (http://geneontology.org/) (accessed on 5 September 2023). The phylogenetic classification of protein families was determined using the Cluster of Orthologous Groups (COG) database (http://www.ncbi.nlm.nih.gov/COG) (accessed 1 September 2023)).

### 4.3. Analysis of the Bioactivity of Microbial Metabolites

To measure IAA production, BJ3 was cultured for 24, 48, and 72 h in Luria Broth (LB) medium supplemented with 2 mg/mL L-tryptophan. Supernatants from the bacterial cultures were added to Salkowski reagent and incubated in the dark at room temperature for 20 min. The absorbance at 535 nm was determined, and the IAA concentration (in ppm) was calculated based on a standard curve derived from known IAA concentrations. The phosphate-solubilizing activity of BJ3 was determined by following procedures described by Nautiyal (1999) [138]. BJ3 was inoculated on the NBRIP plate containing 1% glucose, 0.5% Ca_3_(PO_4_)_2_, 0.5% MgCl_2_·6H_2_O, 0.025% MgSO_4_·7H_2_O, 0.02% KCl, and 0.01% (NH_4_)_2_SO_4_, pH 7.0, and cultured for 14 days. The nitrogen-fixation activity of BJ3 was analyzed on Jensen’s media [139] containing × 25 µg/mL of bromothymol blue. After 4 days of culture, the formation of a yellow zone surrounding the bacterial culture was used as an indicator of nitrogen fixation. The detection of antifungal activity in the metabolites produced by the isolated bacterial strain was carried out using methods outlined by Tsai et al. (2023) [140]. Briefly, two filter paper pieces, each soaked with 10 μL of bacterial solutions at a concentration of 1 × 10^8^ CFU/mL, were placed 3 cm away from a mycelial plug of *Fusarium oxysporum* f. sp. *cubense* tropical race 4 (*Foc* TR4) on potato dextrose agar (PDA) plates. Water served as the control. The cultures were then incubated for seven days at 28 °C. The growth inhibition rate was assessed by measuring the diameter of fungal mycelia in the control and treated groups and calculating the inhibition rate (I) = (1 − diameter of mycelia of treatment/diameter of mycelia of control) × 100.

### 4.4. Analysis of Growth-Promotion Effects 

Seeds of *Arabidopsis thaliana* (ecotype Columbia) were germinated on ½ MS medium for 3 days. Seedlings were transferred to a fresh medium without and with bacterial inoculation and cultured for an additional 4 days. Lateral root number per seedling was estimated. A bacterial culture with a density of 1 × 10^8^ CFU/mL was used to treat *Arabidopsis* seedlings that had been germinating in soil for one week. Each pot contained ten seedlings, and four pots in total were subjected to the bacterial treatment, which was performed weekly over a three-week period. After three treatments, the fresh weight and floral inflorescence of the seedlings were recorded. Furthermore, both the lignin content and the activity of antioxidant enzymes, including ascorbate peroxidase (APX) and guaiacol peroxidase (POD), were analyzed, following the procedures described by Chang et al. [141].

### 4.5. Analysis of Drought and Salt Stress Tolerance

Two-week-old seedlings were treated with BJ3 at a bacterial density of 1 × 10^8^ CFU/mL and then subjected to a water deprivation period of five days. Plants that did not wilt, indicated by stars, were counted. The survival rate was calculated by dividing the number of unwilted plants by the total number of plants. Seven days after the recovery of the water supply, the fresh weight of the seedlings was measured.

For the analysis of salt stress tolerance, after the BJ3 treatment, 125 mM NaCl (30 mL) was applied to both the control and BJ3-treated seedlings. The salt stress treatment was administered three times a week. After two weeks of salt stress treatment, both chlorophyll content and H_2_O_2_ content were measured by following the procedures described by Kurniawan and Chuang (2022) [142].

### 4.6. RNA-Seq Transcriptome Analysis

The leaf tissues from 2-week-old Arabidopsis seedlings treated with BJ3 were harvested for total RNA extraction using the method described by Parcy et al. [143]. Five μg of total RNA was used to construct the RNA-seq library following Illumina’s protocols. After sequencing the library on the Illumina NextSeq 500 platform, the resulting sequences were analyzed using procedures outlined by Tsai et al. (2023) [140]. Gene expression levels were quantified using FPKM (Fragments Per Kilobase of exons per Million mapped reads) values. The fold change (FC) in gene expression was calculated by dividing the expression levels in the treatment group by those in the control group. Genes with a log_2_(FC) greater than 1.0 were considered upregulated; furthermore, genes with a log_2_(FC) equal to or greater than 2.0 were selected for further study.

### 4.7. Statistical Analysis

The differences among the treatments were evaluated using the ANOVA technique within the SAS (3.8) software. Statistical significance was established at a *p*-value of less than 0.05 from the Tukey test.

## 5. Conclusions

The BJ3 genome contains four key gene groups crucial for synthesizing IAA, NRPS, terpenoids, and osmolytes, along with a gene cluster associated with nitrogen fixation. BJ3 produces substantial amounts of IAA and antifungal metabolites while demonstrating nitrogen-fixing and phosphate-solubilizing capabilities. It promotes plant growth by strengthening root architecture, encouraging vegetative growth, and inducing earlier flowering. Additionally, BJ3-treated plants exhibit increased antioxidant activity and reduced negative effects from drought and salt stress. The transcriptome analysis indicates that BJ3 is a potent elicitor of gene expression involved in plant hormone signaling pathways, including auxin, ethylene, and SA. These activated pathways may regulate plant growth, disease resistance, and adaptive responses to abiotic stress.

## Figures and Tables

**Table 1:** BLAST search results.

Bacterial Strains	Sequence Length (bp)	Identity (%)
*Burkholderia contaminans* strain ILQ216	1420	100
*Burkholderia contaminans* strain XL73	1337	100
*Burkholderia* sp. strain GB13	1365	100
*Burkholderia contaminans* strain SK875	1337	100
*Burkholderia* sp. strain NFB35	1417	100
*Burkholderia* sp. strain BC	1378	100
*Burkholderia* sp. strain 337D	1377	100
*Burkholderia* sp. strain 60H	1368	100
*Burkholderia* sp. strain 40H	1374	100
*Burkholderia* sp. strain 1H	1375	100
*Burkholderia cenocepacia* strain PC184	1338	100

**Table 2:** Genes related to plant-growth-promoting traits.

Accession No.	Gene Name	Accession No.	Gene Name
IAA synthesis			
WP_039362343	*trpC*	WP_046196360	*iaaH*
WP_039346423	*IOR*	WP_039366865	*IOR*
WP_039358309	*trpB*	WP_039358314	*trpA*
WP_039356730	*nthB*	WP_039356728	*nthA*
WP_027788472	*trpD*		
Ethylene synthesis			
WP_039371479	*ACC deaminase*		
PQQ synthesis			
WP_039369440	*PqqD*	WP_039369437	*PqqC*
WP_039369434	*PqqB*	QFR11588	*PqqE*
Nitrogen fixation			
WP_011352562	*IscU*, *nifU* homolog	WP_039366622	*Nif3-like*
WP_069586144	*sigma 54-interacting*	WP_039364811	*glnA*
WP_039369321	*glnA*	WP_039355335	*glnA*
WP_039342705	*type I methionyl aminopeptidase*		
Siderophore synthesis			
WP_039358638	*PvdF*		
NRPS/PKS			
WP_039359006	*NRPSs*	WP_039358647	*NRPSs*
WP_039366349	*NRPSs*	WP_039366347	*NRPSs*
WP_039364197	*NRPSs*	WP_039361298	*PksD*
WP_039361196	*DsbA*_*FrnE*		
Hydrolytic enzymes			
MBA9830528	*chitinase*	BBA41828	*cellulase*
VOCs			
-Terpenoid synthesis			
WP_039342858	*ispF*	WP_011352390	*ispD*
WP_039342723	*Dxr*	WP_039360848	*terpene synthase*
-Acetoin biosynthesis			
WP_039351048	*Bdh1*	WP_039344544	*Bdh1*
Lipopolysaccharide synthesis			
WP_039360609	*waaF*	WP_039360073	*LptD*
Spermidine synthesis			
WP_039360784	*speD*	WP_039370372	*SpdS*
WP_039353965	*speB*	WP_039360775	*SPMS*
WP_039339273	*SPMS*		
Osmolytes synthesis			
-Proline			
WP_039361374	*proC*	WP_039353092	*proB*
WP_039361497	*proA*		
-Trehalose			
WP_039348314	*otsB*	WP_039348312	*otsA*
-Glycine betaine			
WP_039369516	*betA*	WP_039354575	*betA*
-Ectoine			
WP_039344448	*ectC*		

## Data Availability

Data is contained within the article and Supplementary Materials.

## Supplementary Materials

The following supporting information can be downloaded at https://www.mdpi.com/article/10.3390/ijms25116091/s1.
