# Supplementary material for: Employing Genomic Tools to Explore the Molecular Mechanisms behind the Enhancement of Plant Growth and Stress Resilience Facilitated by a Burkholderia Rhizobacterial Strain
Source: Int J Mol Sci. 2024 May 31;25(11):6091. doi: 10.3390/ijms25116091 (PMC11172717; doi:10.3390/ijms25116091)
Supplement: Supplementary file 1 [file ijms-25-06091-s001.zip › ijms-3013868-supplementary.pdf]

Table S1. BJ3 upregulated genes

Gene	log2(FC)	TAIR ID	Description
**vegetative growth**			
root, shoot, trichome growth			
CCD7	2.1	AT2G44990	carotenoid cleavage dioxygenase 7
ACR4	3.3	AT5G47850	Serine/threonine-protein kinase-like protein CCR4, CRINKLY4 RELATED 4
EPFL2	3.4	AT4G37810	EPIDERMAL PATTERNING FACTOR-like protein 2
CYCP3;1	2.8	AT2G45080	Cyclin-U2-1
CYCP2;1	2.0	AT3G21870	Cyclin-U1-1
RGP4	2.5	AT5G50750	Probable UDP-arabinopyranose mutase 4
TBL10	2.3	AT3G06080	Protein trichome birefringence-like 10
TBL43	2.0	AT2G30900	Protein trichome birefringence-like 43
SOK2	2.2	AT5G10150	SOK2
RIC7	2.1	AT4G28556	CRIB domain-containing protein RIC7, effector of active Rop2 GTPase
CLE20	2.8	AT1G05065	CLAVATA3/ESR (CLE)-related protein 20
CLE42	2.5	AT2G34925	CLAVATA3/ESR (CLE)-related protein 42
CLE21	2.4	AT5G64800	CLAVATA3/ESR (CLE)-related protein 21
CLE22	2.0	AT5G12235	CLAVATA3/ESR (CLE)-related protein 22
CUC3	2.9	AT1G76420	Protein CUP-SHAPED COTYLEDON 3
DOF1	2.5	AT1G51700	Dof zinc finger protein DOF1.7
MYB39	2.1	AT4G17785	Transcription factor MYB39
XBAT34	2.9	AT4G14365	Putative E3 ubiquitin-protein ligase XBAT34
FRO5	2.1	AT5G23990	ferric reduction oxidase 5, ATFRO4/FRO4 (FERRIC REDUCTION OXIDASE 4)
BT5	2.1	AT4G37610	BTB/POZ and TAZ domain-containing protein 5, transcription regulator
RTFL13	2.4	AT3G23635	ROTUNDIFOLIA like 13
RTFL14	2.2	AT3G63088	ROTUNDIFOLIA like 14
RTFL19	2.0	AT3G02493	ROTUNDIFOLIA like 19
LBD1	2.2	AT1G07900	LOB domain-containing protein 1
LBD11	2.5	AT2G28500	LOB domain-containing protein 11
LBD22	2.0	AT3G13850	LOB domain-containing protein 22
COR27	3.3	AT5G42900	cold regulated gene 27
transporter			
NRT2.5	4.9	AT1G12940	High affinity nitrate transporter 2.5
AMT1;1	2.7	AT4G13510	Ammonium transporter 1 member 1
DUR3	2.0	AT5G45380	Urea-proton symporter DUR3
PTR3	2.2	AT5G46050	PEPTIDE TRANSPORTER 3
OPT1	2.5	AT5G55930	Oligopeptide transporter 1
LHT7	2.5	AT4G35180	Lysine histidine transporter-like 7
SIAR1	2.1	AT1G44800	Siliques Are Red 1, a bidirectional amino acid transporter
AtCAT1	3.3	AT4G21120	Cationic amino acid transporter 1
PHT3;3	2.0	AT2G17270	phosphate transporter 3;3
HAK5	2.2	AT4G13420	Potassium transporter 5
ZIP11	2.8	AT1G55910	Zinc transporter 11
MSS1	3.5	AT5G26340	Sugar transport protein 13

SULTR3;3	2.0	AT1G23090	Probable sulfate transporter 3.3
COPT2	2.3	AT3G46900	Copper transporter 2
**reproduction**			
AP1	3.7	AT1G69120	MADS, Floral homeotic protein APETALA 1
NIP7;1	2.3	AT3G06100	a gated boric acid channel in developing anthers
IDL2	2.7	AT5G64667	Protein IDA-LIKE 2
IDL4	2.8	AT3G18715	Protein IDA-LIKE 4
IDA	2.1	AT1G68765	Protein IDA
CO	3.0	AT5G15840	Zinc finger protein CONSTANS
LFY	2.1	AT5G61850	Protein LEAFY
SPL4	3.1	AT1G53160	Squamosa promoter-binding-like protein 4
SPL13A	2.5	AT5G50570	Squamosa promoter-binding-like protein 13A, anther development,
CYP715A1	2.0	AT5G52400	cytochrome P450, family 715, subfamily A, polypeptide 1
LEA	4.6	AT4G23610	Late embryogenesis abundant (LEA) hydroxyproline-rich glycoprotein family
LEA	2.4	AT3G15670	Late embryogenesis abundant protein (LEA) family protein
Oleosin	3.8	AT5G07571	Oleosin family protein
FLOT1	3.2	AT5G25250	Flotillin-like protein 1, FLOT1
SOM	2.0	AT1G03790	Zinc finger CCCH domain-containing protein 2
GDH1	2.0	AT5G18170	Glutamate dehydrogenase 1
SUC5	3.4	AT1G71890	Sucrose transport protein SUC5
WOX2	2.1	AT5G59340	WUSCHEL-related homeobox 2
**chloroplast function**			
RPI2	2.0	AT2G01290	Probable ribose-5-phosphate isomerase 2
LHB1B1	2.0	AT2G34430	light-harvesting chlorophyll-protein complex II subunit B1
NPQ7	2.5	AT1G65420	Ycf20-like protein, involved in nonphotochemical quenching
CRR7	2.3	AT5G39210	chlororespiratory reduction 7, chloroplastic NAD(P)H dehydrogenase
PGL1	2.3	AT1G13700	6-PHOSPHOGLUCONOLACTONASE 1
FKBP13	2.0	AT5G45680	Peptidyl-prolyl cis-trans isomerase FKBP13, chloroplastic
**cell wall components**			
CCOAMT	4.5	AT1G67980	Putative caffeoyl-CoA O-methyltransferase
CCR2	2.9	AT1G80820	Cinnamoyl-CoA reductase 2
CAD2	2.4	AT2G21730	Cinnamyl alcohol dehydrogenase 2
CAD family	2.4	AT1G09500	NAD(P)-binding Rossmann-fold superfamily protein
EXLA3	3.2	AT3G45960	Expansin-like A3
EXLA2	2.0	AT4G38400	Expansin-like A2
CSLB03	3.9	AT2G32530	Cellulose synthase-like protein B3
AGP5	2.9	AT1G35230	Classical arabinogalactan protein 5
AGP6	2.6	AT5G14380	Classical arabinogalactan protein 6
GALK2	3.4	AT5G14470	Probable glucuronokinase 2
EXL5	2.0	AT2G17230	Protein EXORDIUM-like 5
LACS3	2.0	AT1G64400	Long chain acyl-CoA synthetase 3
peroxidase 4	3.2	AT1G14540	Peroxidase 4
Peroxidase 71	2.5	AT5G64120	Peroxidase 71
MYB87	2.8	AT4G37780	myb domain protein 87

MYB15	3.1	AT3G23250	myb domain protein 15
**hormone signaling**			
auxin			
UGT74E2	5.6	AT1G05680	UDP-glycosyltransferase 74E2
YUC5	3.0	AT5G43890	Probable indole-3-pyruvate monooxygenase YUCCA5
MYB77	3.3	AT3G50060	myb domain protein 77
NIT2	2.7	AT3G44300	Nitrilase 2
GH3 protein	2.4	AT1G48660	Auxin-responsive GH3 family protein
GH3.1	2.0	AT2G14960	Probable indole-3-acetic acid-amido synthetase GH3.1
GH3.12	3.3	AT5G13320	4-substituted benzoates-glutamate ligase GH3.12
SAUR71	4.1	AT1G56150	Auxin-responsive protein SAUR71
SAUR48	3.4	AT3G09870	SAUR-like auxin-responsive protein family
SAUR72	3.2	AT3G12830	Auxin-responsive protein SAUR72
SAUR41	2.4	AT1G16510	Auxin-responsive protein SAUR41
SAUR45	2.3	AT2G36210	SAUR-like auxin-responsive protein family
SAUR5	2.2	AT4G34810	SAUR-like auxin-responsive protein family
SAUR4	2.0	AT4G34800	SAUR-like auxin-responsive protein family
Auxin-resp.	2.0	AT5G35735	Cytochrome b561 and DOMON domain-containing protein
PIN-LIKES 7	2.0	AT5G65980	Auxin efflux carrier family protein
ethylene			
ACS6	3.9	AT4G11280	1-aminocyclopropane-1-carboxylate synthase 6
ACS11	2.6	AT4G08040	1-aminocyclopropane-1-carboxylate synthase 11
ACS7	3.1	AT4G26200	1-aminocyclopropane-1-carboxylate synthase 7
SA			
CBP60G	2.9	AT5G26920	"Calmodulin-binding protein 60 G, CBP60G, SA
SARD1	2.0	AT1G73805	Protein SAR DEFICIENT 1
ABA			
NCED9	2.8	AT1G78390	9-cis-epoxycarotenoid dioxygenase NCED9, chloroplastic
ARCK1	6.6	AT4G11890	Cysteine-rich receptor-like protein kinase 45, ARCK1
PP2CA	2.1	AT3G11410	Protein phosphatase 2C 37
cytokinin			
IPT7	3.4	AT3G23630	Adenylate isopentenyltransferase 7
RR16	2.7	AT2G40670	Two-component response regulator ARR16
ARR15	4.3	AT1G74890	Two-component response regulator ARR15
CKX7	2.0	AT5G21482	Cytokinin dehydrogenase 7
UGT85A1	2.6	AT1G22400	UDP-glycosyltransferase 85A1
GA			
GASA1	2.0	AT1G75750	Gibberellin-regulated protein 1
GA3OX2	2.0	AT1G80340	Gibberellin 3-beta-dioxygenase 2
ATGA2OX1	2.0	AT1G78440	Gibberellin 2-beta-dioxygenase 1
GA20OX3	2.0	AT5G07200	Gibberellin 20 oxidase 3
abiotic stress			
DIC2	5.1	AT4G24570	Mitochondrial uncoupling protein 4

TSPO	2.0	AT2G47770	Translocator protein homolog
ADC2	2.1	AT4G34710	Arginine decarboxylase 2
ADC1	2.0	AT2G16500	Arginine decarboxylase 1
OCT5	3.4	AT1G79410	Organic cation/carnitine transporter 5
CML37	2.6	AT5G42380	Calcium-binding protein CML37
CRK5	2.6	AT4G23130	Cysteine-rich receptor-like protein kinase 5
PROT2	2.0	AT3G55740	Proline transporter 2
ProT3	2.2	AT2G36590	Proline transporter 3
RAS1	3.1	AT1G09950	RESPONSE TO ABA AND SALT 1
STZ	2.9	AT1G27730	Zinc finger protein ZAT10
SLAH1	2.5	AT1G62280	S-type anion channel SLAH1
UGT85A5	2.1	AT1G22370	UDP-glycosyltransferase 85A5
NAS2	4.3	AT5G56080	Nicotianamine synthase 2
ALMT1	3.6	AT1G08430	Aluminum-activated malate transporter 1
CZF1	2.2	AT2G40140	Zinc finger CCCH domain-containing protein 29
COR413IM1	2.7	AT1G29395	Cold-regulated 413 inner membrane protein 1
COR314-TM2	2.0	AT1G29390	Cold-regulated 413 inner membrane protein 2
ATGLR1.2	2.0	AT5G48400	Glutamate receptor 1.2
MYB47	2.5	AT1G18710	myb domain protein 47
CHS1	2.3	AT1G17610	CHILLING SENSITIVE 1
Disease resistance			
induced immunity			
FRK1	6.4	AT2G19190	Senescence-induced receptor-like serine/threonine-protein kinase
PP2-A5	4.8	AT1G65390	Protein PHLOEM PROTEIN 2-LIKE A5
MPK11	3.3	AT1G01560	Mitogen-activated protein kinase 11
LECRKA4.1	2.7	AT5G01540	L-type lectin-domain containing receptor kinase VI.2
AtCMPG1	2.4	AT1G66160	U-box domain-containing protein 20
CRK4	2.7	AT3G45860	Cysteine-rich receptor-like protein kinase 4
CRK6	4.2	AT4G23140	Cysteine-rich receptor-like protein kinase 6
CRK7	2.9	AT4G23150	Cysteine-rich receptor-like protein kinase 7
CRK20	4.0	AT4G23280	cysteine-rich RLK (RECEPTOR-like protein kinase) 20
CRK13	2.6	AT4G23210	Cysteine-rich receptor-like protein kinase 13
CRK10	2.0	AT4G23180	Cysteine-rich receptor-like protein kinase 10
Disease resistance proteins			
ACD6	4.6	AT4G14400	ACCELERATED CELL DEATH 6
AtPME41	3.8	AT4G02330	pectin methylesterase 41
NDR1	2.6	AT3G20600	Protein NDR1
RD19	2.2	AT2G21430	Probable cysteine proteinase A494
HR3	2.1	AT3G50470	RPW8-like protein 3
MLO12	2.3	AT2G39200	MLO-like protein 12
AIG2	2.2	AT3G28930	Protein AIG2
PDLP1	2.9	AT5G43980	Cysteine-rich repeat secretory protein 56
GLIP1	2.0	AT5G40990	GDSL esterase/lipase 1
SIB1	2.4	AT3G56710	Sigma factor binding protein 1

MPL1	2.2	AT5G14180	Triacylglycerol lipase 2
RLP23	2.6	AT2G32680	receptor like protein 23
RLP53	2.1	AT5G27060	receptor like protein 53
GAD4	2.1	AT2G02010	Glutamate decarboxylase 4
NIMIN1	2.1	AT1G02450	Protein NIM1-INTERACTING 1
PR genes			
PR1	2.2	AT2G14610	Pathogenesis-related protein 1
CHI	2.1	AT2G43570	chitinase, putative
PDF1.3	4.9	AT2G26010	Defensin-like protein 14
PDF1.4	3.4	AT1G19610	Defensin-like protein 19
PDF2.1	3.3	AT2G02120	Defensin-like protein 4
DEFLs	2.9	AT5G44973	Defensin-like protein 285
PDF1.2c	2.9	AT5G44430	Defensin-like protein 17
KPI1-2	2.9	AT4G01575	serine protease inhibitor
PI	2.2	AT2G38870	Serine protease inhibitor, PR-6 proteinase inhibitor
SERPIN	2.1	AT2G35580	Serine protease inhibitor (SERPIN) family protein, proteolysis
TLP-3	2.7	AT1G19320	Pathogenesis-related thaumatin superfamily protein
metabolites			
glucosinolate			
IGMT2	4.7	AT1G21120	O-methyltransferase family protein
CYP81F2	2.3	AT5G57220	cytochrome P450, family 81, subfamily F, polypeptide 2
GTR1	2.0	AT3G47960	GLUCOSINOLATE TRANSPORTER-1
camalexin			
PAD3	4.2	AT3G26830	Bifunctional dihydrocamalexate synthase/camalexin synthase
CYP71A12	3.4	AT2G30750	cytochrome P450, family 71, subfamily A, polypeptide 12
cyanogenic phytoalexin			
FOX1	5.3	AT1G26380	ATBBE3, FOX1
terpenoid			
BARS1	4.4	AT4G15370	Baruol synthase
Terp. cyclases	2.0	AT1G48820	Terpenoid cyclases/Protein prenyltransferases superfamily protein
CYP705A3	3.1	AT4G15360	CYP705A3
CYP76C2	2.1	AT2G45570	Cytochrome P450 76C2
**transcription factors**			
CBF1	5.5	AT4G25490	DRE BINDING PROTEIN 1B
CBF4	3.4	AT5G51990	DREB1D
CBF2	2.4	AT4G25470	DREB1C
RRTF1	4.2	AT4G34410	RRTF1
ERF13	4.6	AT2G44840	Ethylene-responsive transcription factor 13
ERF017	4.0	AT1G19210	Ethylene-responsive transcription factor ERF017
ERF114	3.7	AT5G61890	Ethylene-responsive transcription factor ERF114
ERF61	3.5	AT1G64380	Ethylene-responsive transcription factor ERF061
ERF043	3.1	AT4G32800	Ethylene-responsive transcription factor ERF043
ERF016	2.9	AT5G21960	Ethylene-responsive transcription factor ERF016
ERF104	2.4	AT5G61600	Ethylene-responsive transcription factor ERF104

ERF116	2.2	AT1G25470	Ethylene-responsive transcription factor ERF116
ERF019	2.0	AT1G22810	Ethylene-responsive transcription factor ERF019
ERF39	2.0	AT4G16750	Ethylene-responsive transcription factor ERF039
SHN2	2.1	AT5G25390	Ethylene-responsive transcription factor SHINE 3
CRF1	2.0	AT4G11140	Ethylene-responsive transcription factor CRF1
ddf2	2.1	AT1G63030	Dehydration-responsive element-binding protein 1E
DDF1	4.3	AT1G12610	Dehydration-responsive element-binding protein 1F
DREB	3.4	AT2G40350	DREB subfamily A-2 of ERF/AP2 transcription factor
WRKY30	4.1	AT5G24110	Probable WRKY transcription factor 30
WRKY71	3.9	AT1G29860	Probable WRKY transcription factor 71
WRKY53	3.3	AT4G23810	Probable WRKY transcription factor 53
ABO3	3.1	AT1G66600	Probable WRKY transcription factor 63
WRKY43	2.1	AT2G46130	Probable WRKY transcription factor 43
WRKY48	2.0	AT5G49520	Probable WRKY transcription factor 48
SZF1	2.5	AT3G55980	CCCH domain-containing protein 47
